# A Spectrum of Biopsy – Proven Renal Disorders and Their Clinicopathological Correlation in Elderly Population From a Tertiary Care Center in South India

**DOI:** 10.7759/cureus.17031

**Published:** 2021-08-09

**Authors:** Josephine S, Barathi G, Susruthan M, Subalakshmi Balasubramanian

**Affiliations:** 1 Pathology, Sri Ramachandra Institute of Higher Education and Research, Chennai, IND

**Keywords:** chronic kidney disease, elderly, renal biopsy, egfr, glomerular diseases

## Abstract

Introduction

Chronic kidney disease (CKD) has become a health concern with an extensive burden on incidence and prognosis. While the increasing lifespan contributes to a higher incidence of CKD among the elderly, the diagnostic picture in this age group is complicated by senescence-related changes. A better understanding of the etiology and progression of the disease warrants renal biopsy in such patients. This study aims to explore the histopathological spectrum of native renal biopsies leading to CKD in elderly patients in a tertiary care hospital.

Methods

Among the list of patients who had undergone renal biopsy at our institute from January 2015 to March 2020, elderly patients aged ≥ 60years were chosen for this study. Their demographic details, lab investigations and histopathological reports were collected. The sex distribution and prevalence of different renal diseases was calculated. The subjects were classified into four broad diagnostic groups - primary glomerular disease, diabetic nephropathy, hypertensive nephropathy, and tubulointerstitial disease. The estimated glomerular filtration rate (eGFR) values were calculated and used to stage chronic kidney disease in these patients. Statistical analysis was carried out to find a correlation between diagnostic groups and CKD presence and between serum C3 values and immunofluorescence for the same on biopsy.

Results

One hundred thirty-two patients formed the study sample with a male to female ratio of 1.28:1, showing a slight male predominance. The most common diagnostic group was primary glomerular disease (46%), among which focal segmental glomerulosclerosis (FSGS) was the most common entity (12%). 47.7% and 66.6% of patients in the study sample showed elevated serum blood urea nitrogen (BUN) and creatinine values, respectively. 86% of our study sample had low eGFR values, and the majority (35%) of the patients were classified under CKD stage 3. CKD incidence was high among patients with primary glomerular diseases, but no significant statistical correlation was found. 43.5% of all IF positive cases showed low serum C3 values and established a positive correlation between IF and serum C3 values.

Conclusion

There is no statistically significant correlation of the four diagnostic groups to the CKD. CKD in the elderly may be multifactorial, and a collaborative study across the nations may be needed to further evaluate the etiology.

## Introduction

CKD is a common public health-related issue with a higher incidence rate and poorer prognosis in recent years. It is a financially challenging disease globally and is frequently seen in older adults [1]. As the life span of individuals increases, the patients with acute and chronic kidney disease continue to survive longer [2]. Moreover, the onset of the Covid-19 pandemic has highlighted CKD as a significant co-morbid factor. The risk of severe disease and mortality is attributed to the presence and stages of CKD [3].

In elderly patients, senescence is also the contributing factor along with the wide-scale of renal diseases, which varies with the range of commonly noted renal disorders seen in younger patients. Thus, a definitive diagnosis is not possible due to the intricacy of the ageing process, the clinical conditions, and the morphological manifestations of an individual's disease progression [4]. This subsequently impedes proper treatment planning and the prognosis of the disease. Numerous previous studies have established the factors contributing to CKD onset but have not distinguished patients based on age [5]. Therefore, a better understanding of the etiological factors and the progression of the disease to end-stage renal disease (ESRD) among the elderly is important.

Renal biopsy is unparalleled in diagnosing most kidney disease patients due to the pronounced activity and constancy of the kidney injury. A patient's age is no more considered a contraindication to performing a renal biopsy, immunosuppressive therapy, renal transplant, and renal replacement. Renal biopsy plays a pivotal role in diagnosing and treating renal disorders in aged patients [6]. The present study explores the histopathological spectrum of native renal biopsies leading to CKD in elderly patients (>60 years) in a tertiary care hospital. Furthermore, this study also tests the hypothesis of a significant correlation between the stage of CKD and the underlying renal pathology.

## Materials and methods

Study design

This retrospective study was quantitative and descriptive.

Study population

The list of all patients who have undergone renal biopsy at Sri Ramachandra Medical College, Chennai, India, was retrieved for this study from the LIS (lab information system).

Study period

The study data was collected over five years, from January 2015 to March 2020.

Sample size and sampling

All elderly patients (aged 60 years and above, as defined by National Policy on Older Persons) from the LIS data were chosen for this study. A total of 132 formed the sample size.

Data collection tools

Relevant demographic data (age and sex) was collected for each patient, along with their biopsy reports. Histopathology included light microscopy slides stained with Hematoxylin and Eosin (H and E), Periodic Acid Schiff (PAS), Jones Methanamine Silver (JMS), Masson’s Trichrome (MT) stains. Specimens for immunofluorescence (IF) microscopy were stained using fluorescein isothiocyanate (FITC) conjugated polyclonal rabbit antisera against human IgG, IgM, IgA, C3c, C1q, kappa, lambda, fibrinogen and albumin. IF findings were categorized based on location and intensity of staining from (+) to (++++).

Further, reports were collected for few biochemical (BUN - Blood Urea Nitrogen, Creatinine) and other tests, especially urine analysis done around the time of renal biopsy. The inclusion criteria for this study was based on the availability of demographic data, histopathology reports and biochemistry test results. Biopsies from subjects less than 60 years of age and transplant recipients, as well as those considered inadequate for reporting were excluded from this study.

Ethical approval and informed consent

Permission from the Institutional Ethics Committee (IEC) was obtained before commencing this study. IEC reference ID is given here. (REF: CSP-MED/20/SEP/61/88).

Operational definition

The demographic data of the patients was analyzed to determine the sex distribution of the study sample. Their histopathological diagnosis was used to classify these subjects into four broad diagnostic groups - primary glomerular disease, diabetic nephropathy, hypertensive nephropathy, and tubulointerstitial disease. The prevalence of different renal diseases was calculated. The age and serum creatinine values were used in the MDRD (Modification of Renal Diet) equation to calculate the estimated glomerular filtration rate (eGFR) value for each of these patients, which was then plotted on a graph. The eGFR value was also used in detection and grading of CKD in these patients. All patients were categorized into different stages of CKD based on their eGFR values, using the National Kidney Foundation criteria.

Statistical analysis

The incidence of CKD was compared among the different diagnostic groups mentioned above. Statistical analysis was carried out using the SPSS software. Chi-square test was used to check for presence of correlation among the variables. Serum C3 levels were used for clinicopathological correlation with IF positivity for C3, and their correlation was established using the T-test.

## Results

**Figure 1:**
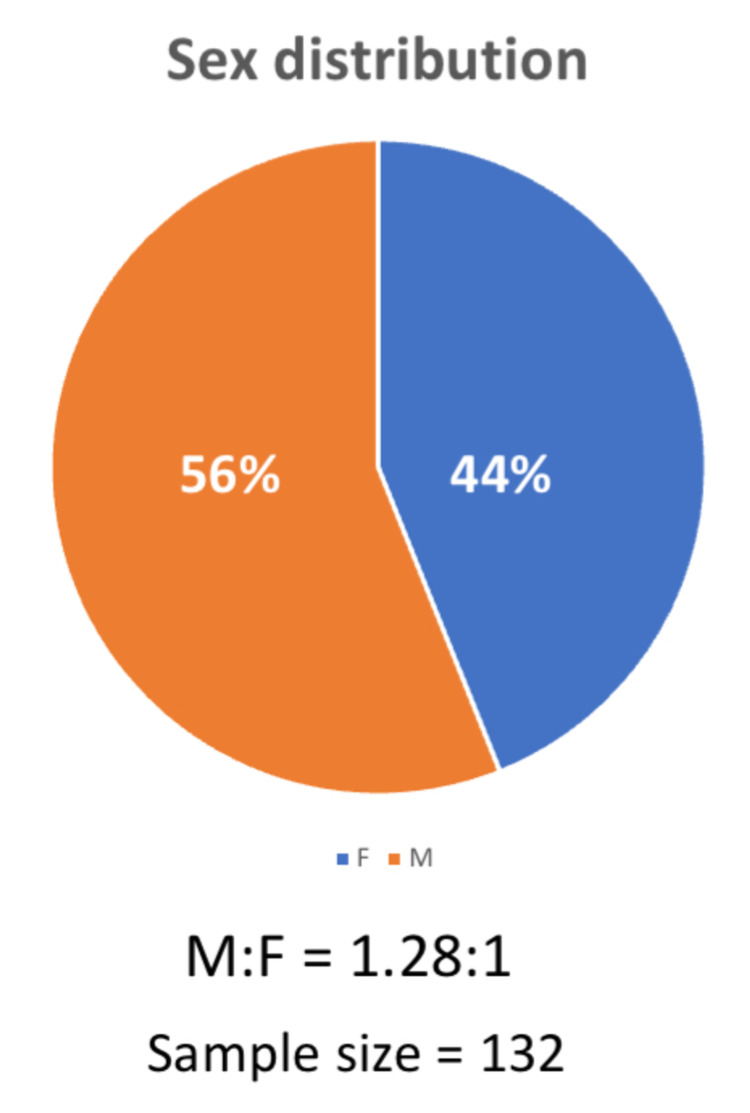
Gender distribution of the study sample showing a slight male predominance M- Male; F- Female

The total number of renal biopsies received in our department during our study period (January 2015 to March 2020) was 946, of which 132 (13.9%) were from elderly patients (Age ≥ 60 years). Our study sample's male to female ratio was 1.28:1, showing a slight male predominance (Fig 1).

**Figure 2:**
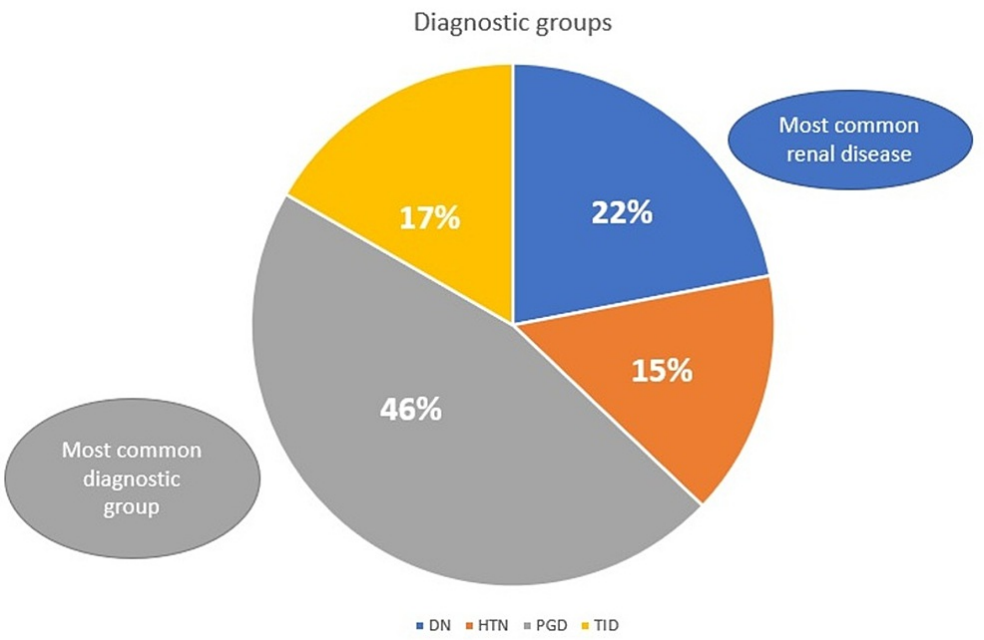
Categorization of study subjects into four diagnostic groups. PGD- Primary glomerular disease, DN- Diabetic nephropathy, HTN- Hypertensive nephropathy, TID- Tubulo-interstitial disease

After classification of cases into the four diagnostic groups, it was observed that the most common among them was a primary glomerular disease, accounting for almost half the patients in our study sample (46%) (Fig 2).

On sub-classification among the glomerular diseases, it was seen that the most common primary glomerular disease is Focal Segmental Glomerulosclerosis (FSGS) which accounted for 12% of all cases (Table 1).

**Table 1:** Sub-classification of various primary glomerular diseases with lab parameters for CKD staging FSGS- Focal Segmental Glomerulosclerosis, IRGN- Infection-related glomerulonephritis, MGN- Membranous glomerulonephritis, MCD- Minimal change disease, DPGN- Diffuse proliferative glomerulonephritis, IgAN- IgA nephropathy, Crescentic GN- Crescentic glomerulonephritis, LN- Lupus nephritis, TMA- Thrombotic microangiopathy, MPGN- Membranoproliferative glomerulonephritis

Glomerular diseases	Frequency (%)	Creatinine	eGFR	CKD grade	Urine protein
FSGS	16 (12%)	0.6-2.8	12.14-148.67	1-5	0-4+
IRGN	15 (11.3%)	0.6-5.1	12.53-110.32	1-5	0-4+
MGN	10 (7.5%)	0.3-3.3	15.37-243.71	1-4	1+-4+
MCD	7 (5.3%)	0.5-2.9	17.65-134.69	1-4	3+-4+
DPGN	6 (4.5%)	0.5-2.7	20.74-127.84	1-4	trace-3+
IgAN	4 (3%)	1.5-4.3	15.05-48.76	3-4	1+-3+
Crescentic GN	3 (2.2%)	2.4-5.6	11.29-21.60	4-5	2+-3+
LN	3 (2.2%)	0.9-4.3	11.37-68.11	2-5	3+-4+
Amyloidosis	3 (2.2%)	1.2-2.2	22.94-49.04	3-4	3+-4+
TMA	2 (1.5%)	3.8-4.5	12.97-14.28	5	1+-2+
MPGN	1 (0.7%)	2.5	27.19	4	3+

**Figure 3:**
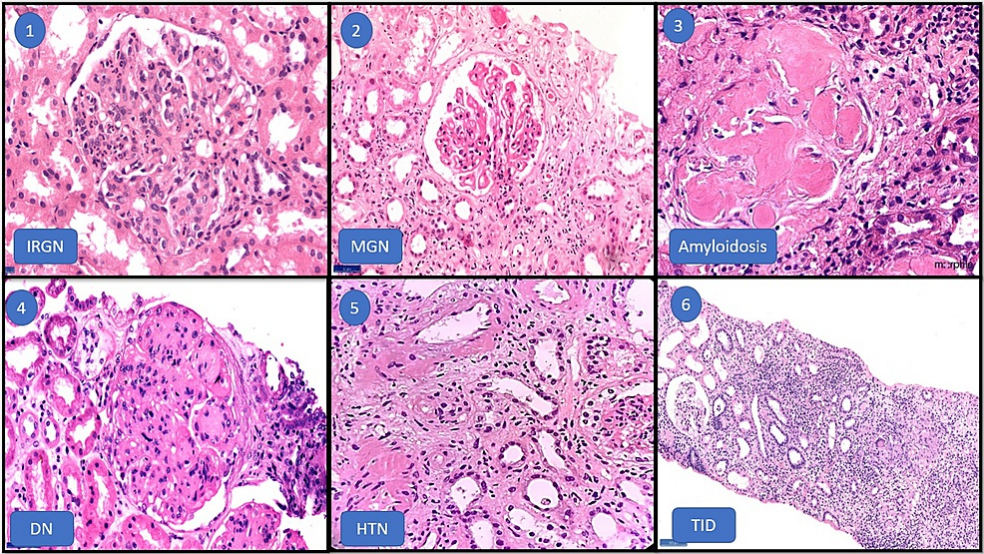
H&E images of the most commonly encountered histopathology diagnoses in the study sample IRGN- Infection-related glomerulonephritis, MGN- Membranous glomerulonephritis, DN- Diabetic nephropathy, HTN- Hypertensive nephropathy, TID- Tubulo-interstitial disease

Other primary glomerular diseases noted were infection-related glomerulonephritis (IRGN) (11.3%), membranous glomerulonephritis (7.5%), minimal change disease (5.3%), diffuse proliferative glomerulonephritis (4.5%), IgA nephropathy (3%), crescentic glomerulonephritis (2.2%), lupus nephritis (2.2%), amyloidosis (2.2%), thrombotic microangiopathy (1.5%) and membranoproliferative glomerulonephritis (0.7%) (Fig 3).

**Figure 4:**
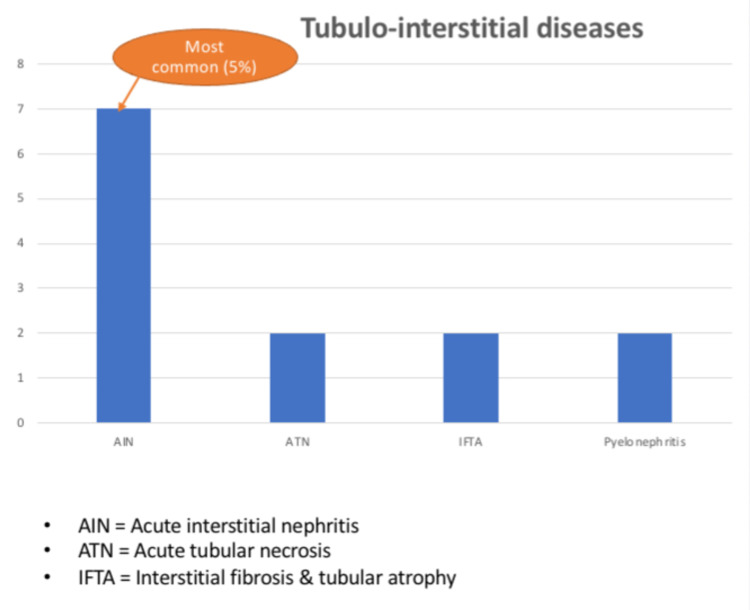
Sub-categorization of tubulointerstitial diseases seen in the study sample

The second most common group was diabetic nephropathy (22%). Tubulointerstitial diseases account for 17% of the cases, and the remaining 15% consisted of cases of hypertensive nephropathy. Among tubulointerstitial diseases, acute interstitial nephritis (5.3%) was the most common diagnosis, and other diagnoses included acute tubular necrosis (1.5%), pyelonephritis (1.5%), and interstitial fibrosis and tubular atrophy (1.5%) (Fig 4).

**Figure 5:**
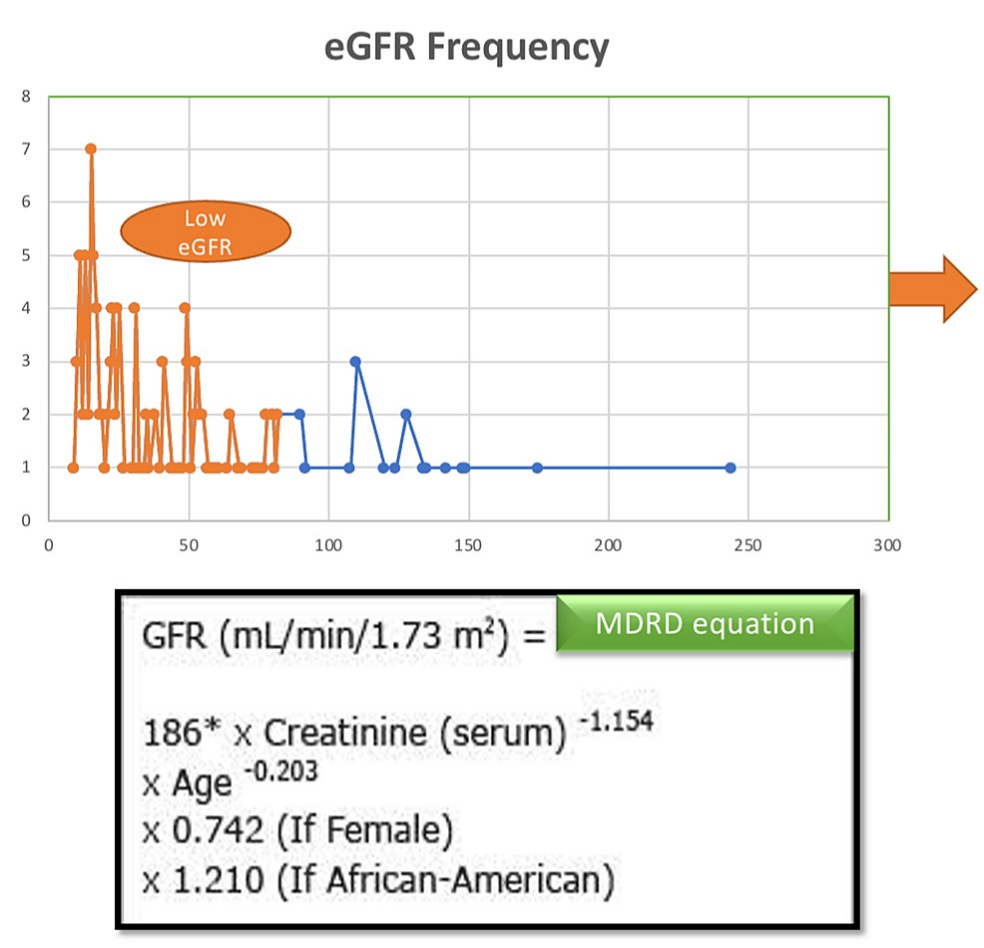
eGFR values (calculated using the MDRD equation) plotted on a graph. The values highlighted in orange indicate the cases with low eGFR, implicating the burden of CKD in the study population. *The MDRD equation image is downloaded from Google.* *eGFR- *estimated glomerular filtration rate; MDRD- Modification of Renal Diet; CKD- Chronic Renal Disease; GFR- Glomerular filtration rate

47.7% and 66.6% of patients in the study sample showed elevated serum BUN and creatinine values, respectively. On plotting the calculated eGFR values of all patients on a graph, it was observed that 86% of our study sample had low eGFR values (Fig 5).

**Figure 6:**
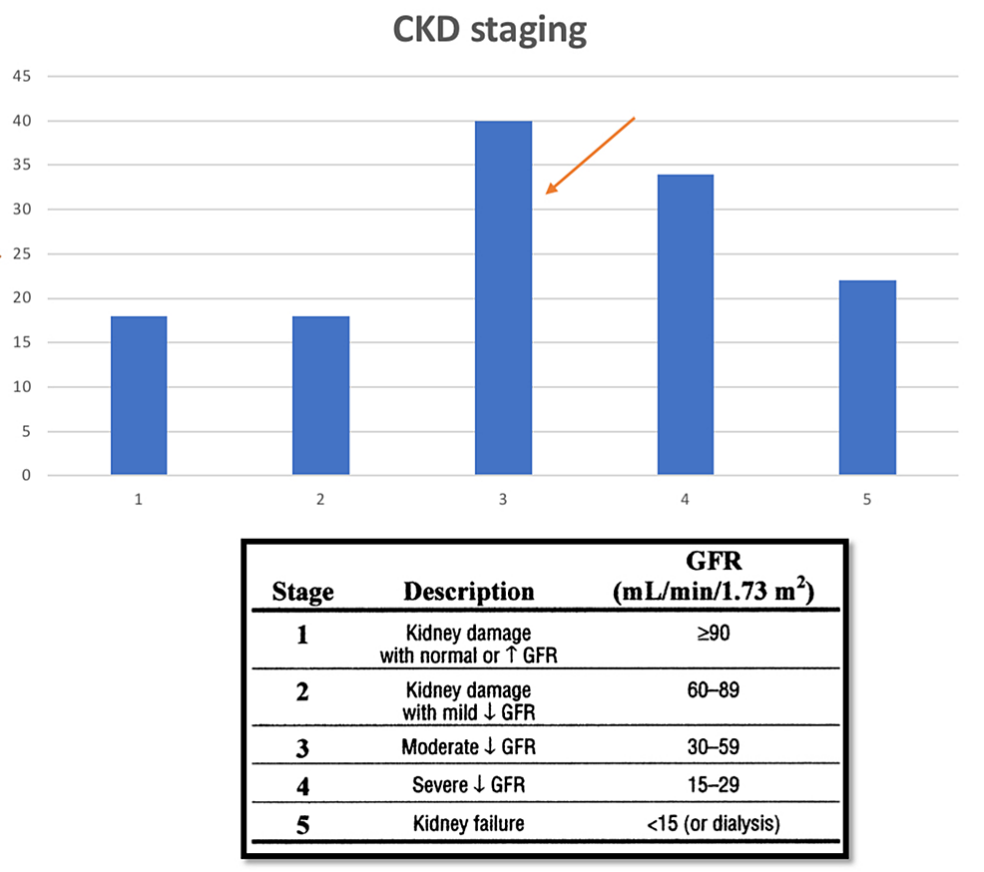
Graph showing the distribution of grades of CKD among the cases in study sample. Grading has been done according to the above-mentioned criteria, using eGFR values calculated on application of the MDRD equation. Majority of the cases in the study sample fall under CKD grade 3. eGFR- estimated glomerular filtration rate; MDRD- Modification of Renal Diet; CKD- Chronic Renal Disease; GFR- Glomerular filtration rate

Among the patients with chronic kidney disease, the majority (35%) of the patients were classified under CKD stage 3 (Fig 6).

**Figure 7:**
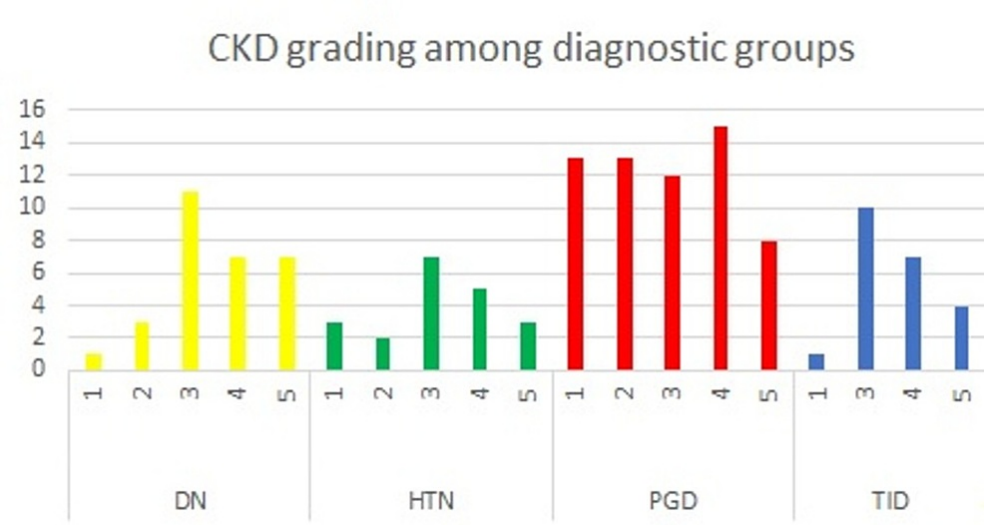
Graph comparing the incidence and grades of CKD amongst the 4 diagnostic groups. The highest incidence of CKD is noted in cases of primary glomerulopathy, amongst whom CKD grade 4 is the most common. DN- Diabetic nephropathy; HTN- Hypertensive nephropathy; TID- Tubulo-interstitial disease; PGD- Primary glomerular disease; CKD- Chronic kidney disease

On comparison among diagnostic groups, it was noticed that the incidence of CKD was high among patients with primary glomerular diseases (Fig 7).

**Figure 8:**
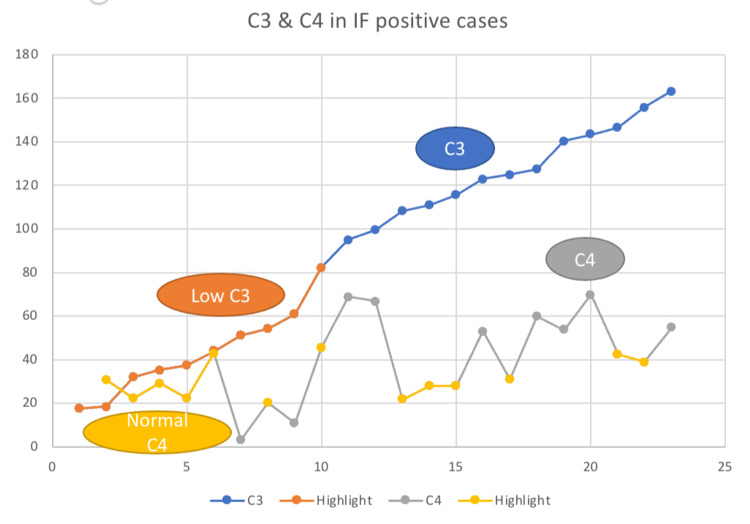
Graph showing the distribution of serum C3 and C4 values among cases which show positive immunofluorescence Serum C3 values highlighted in orange depict the cases showing a clinical correlation between low serum C3 values and positive immunofluorescence for C3 in renal biopsy.

However, applying the chi-square test for analysis of the correlation between diagnostic group and CKD stage, no significant association was noted between these two variables (p-value = 0.094). Similarly, a high incidence of proteinuria was noted in cases of primary glomerular diseases. Serum C3 values of patients with positive IF report for C3 were plotted on a graph (Fig 8).

43.5% of all IF positive cases showed low serum C3 values. On applying the student t-test, a positive correlation was established between IF and serum C3 values (p value= 0.04).

## Discussion

There is a high prevalence of CKD among the elderly. The improvement in life expectancy is associated with an increase in age-related diseases like hypertension, diabetes and CKD. According to the Third National Health and Nutrition Examination Survey (NHANES) data in the US, the presence or absence of CKD can be confirmed by non-typical renal markers such as increased levels of protein or abnormal cells in the urine, atypical radiological findings, etc. Also, a depletion to less than 60 ml/min/1.73m2 in total eGFR for a minimum of 3 months confirms CKD, which may even lead to death.

The incidence of CKD is greatly dependent on age and sex [7]. In the current study, elders constituted 13.9% of all the renal biopsies received during the study period. This observation was similar to other studies which showed an increasing trend in the total number of elders undergoing a renal biopsy, for the obvious reason of increased life expectancy worldwide [8]. In concordance with other studies, our study also showed a slight male predominance (1.28:1) [9].

When analyzed, the spectrum of renal diseases affecting the elderly is not similar to that of young people [10]. However, the clinical and morphological picture in the elderly is made more ambiguous by age-associated changes, making its diagnosis and prognostication difficult. Studies show that the two most common indications for biopsy in the elderly population are acute kidney injury and nephrotic syndrome of rapid onset [11,12]. This was in line with our study where the most common clinical presentation was nephrotic syndrome (68%) followed by acute kidney injury (AKI) (57%). In the primary glomerular diseases, we also found that the serum C3 levels showed a significant correlation with the IF findings.

The four major diagnostic groups in our study include primary glomerular diseases, Diabetic nephropathy, Hypertensive Nephropathy and Tubulointerstitial diseases. The majority of the cases were that of primary glomerular diseases in which FSGS was the most common subgroup observed followed by IRGN. The high prevalence of CKD in primary glomerular diseases can be attributed to the high incidence of this diagnostic group in our study sample (46%).

The second most common diagnosis encountered in the present study was diabetic nephropathy. The NHANES study underlined how diabetes had a stronger impact on renal function than ageing itself, showing that the increasing prevalence of renal impairment in the US population in the period 2005-2008 was related to the increasing trends of diabetes, while the age distribution of that population did not change during the observation period [13]. The overall high prevalence of diabetic kidney disease in elderly T2DM (Type 2 Diabetes Mellitus) patients is likely to be the result of two opposite trajectories, i.e. the high frequency of T2DM in this age group, and the decreased mortality rate due to better control of the disease, which may have contributed to increased survival, allowing sufficient time to develop complications of chronic diabetes, including renal damage.

The third most commonly observed diagnostic group in our study was hypertensive nephropathy. Renal insufficiency in association with hypertension is noted commonly in many studies [14]. Our study also showed a similar pattern of renal injury in the form of rising creatinine or proteinuria, among many patients diagnosed with renal failure secondary to hypertension (58%). Renal vasculitis is more common in the elderly than in the young [1,6]. Only 2.2% of our cases showed crescentic glomerulonephritis.

The fourth diagnostic group is tubulointerstitial diseases causing CKD. In this group, we observed that the most common cause for acute interstitial nephritis in our population is native medication intake, leading to proteinuria and raised creatinine. The worst outcome observed in this category may be attributed to the existing renal damage in an elderly population, secondary to age-related nephron loss and the compromise in GFR. Interstitial fibrosis and tubular atrophy (IFTA) and acute tubular necrosis by themselves can also occur as secondary changes in any primary glomerular diseases. Hence, this diagnostic group does not contribute to the association of the disease to CKD as well.

GFR is considered the best overall measure of renal function. eGFR is estimated using the MDRD equation or the CKD-EPI (CKD-Epidemiology) collaboration formula (initially estimated using creatinine clearance). The value of eGFR, which is used in the detection and staging of CKD, shows a steady decline with age, further increasing the susceptibility to other co-morbidities and drug nephrotoxicity. The staging of CKD helps in guiding the choice of therapy and monitoring the prognosis of the patient [15,16].

The role of eGFR in the estimation of CKD is limited due to the over-diagnosis of CKD (without any other evidence of kidney damage) and the use of multiple formulas in its calculation. However, it is critical in interpreting the signs and symptoms, as well as the laboratory findings which could manifest kidney disease. A study done by BR Hemmelgarn et al. has evaluated the progression of CKD by assessing the decline in eGFR per year and has highlighted diabetes to be an important contributing factor [17]. Our study showed low eGFR in 86% of the subjects, pointing to a high prevalence of CKD among the elderly.

Statistical analysis among the diagnostic groups to establish the causal association of the disease with CKD did not show a significant correlation. This can be attributed to multiple factors like time of presentation of individual cases, diagnostic accuracy and also treatment strategies. In addition, this may be due to several social determinants like geographic distribution of the various renal diseases and lifestyle of the population, which act as confounding factors, contributing to the onset and progression of CKD [18]. Many elderly patients may not be started on immunosuppressive therapy for the fear of infections, which again has an impact on the renal outcome.

## Conclusions

The present study was conducted to establish a causal association of various renal diseases to CKD among the elderly. But on analysis, we found that there is no statistically significant correlation of the four diagnostic groups to CKD. The limitations of this study may be due to its retrospective design and sample size. Because of its diversity of presentation, CKD in the elderly may be multifactorial, and a collaborative study across the nations may be needed to further evaluate the etiology.

## References

[1] Chen L, Luodelete M, Dong C (2019). Pathological spectrum of glomerular disease in patients with renal insufficiency: a single-center study in Northeastern China. Ren Fail.

[2] Perkowska-Ptasinska A, Deborska-Materkowska D, Bartczak A (2016). Kidney disease in the elderly: biopsy based data from 14 renal centers in Poland. BMC Nephrol.

[3] Menon T, Gandhi SA, Tariq W (2021). Impact of chronic kidney disease on severity and mortality in COVID-19 Patients: a systematic review and meta-analysis. Cureus.

[4] Jin B, Zeng C, Ge Y (2014). The spectrum of biopsy-proven kidney diseases in elderly Chinese patients. Nephrol Dial Transplant.

[5] O'Hare AM, Choi AI, Bertenthal D (2007). Age affects outcomes in chronic kidney disease. J Am Soc Nephrol.

[6] Fiorentino M, Bolignano D, Tesar V (2016). Renal biopsy in 2015--from epidemiology to evidence-based indications. Am J Nephrol.

[7] Mallappallil M, Friedman EA, Delano BG, McFarlane SI, Salifu MO (2014). Chronic kidney disease in the elderly: evaluation and management. Clin Pract (Lond).

[8] Brown CM, Scheven L, O'Kelly P, Dorman AM, Walshe JJ (2012). Renal histology in the elderly: indications and outcomes. J Nephrol.

[9] Chen Y, Li P, Cui C, Yuan A, Zhang K, Yu C (2016). Biopsy-proven kidney diseases in the elderly: clinical characteristics, renal histopathological spectrum and prognostic factors. J Int Med Res.

[10] Zhu P, Zhou FD, Zhao MH (2014). The renal histopathology spectrum of elderly patients with kidney diseases: a study of 430 patients in a single Chinese center. Medicine (Baltimore).

[11] Moutzouris DA, Herlitz L, Appel GB, Markowitz GS, Freudenthal B, Radhakrishnan J, D'Agati VD (2009). Renal biopsy in the very elderly. Clin J Am Soc Nephrol.

[12] Thurman JM (2015). Complement in kidney disease: core curriculum 2015. Am J Kidney Dis.

[13] de Boer IH, Rue TC, Hall YN, Heagerty PJ, Weiss NS, Himmelfarb J (2011). Temporal trends in the prevalence of diabetic kidney disease in the United States. JAMA.

[14] Elaine Ku, Benjamin J. Lee, Jenny Wei, and Matthew R (2019). Hypertension in CKD: core curriculum 2019. Am J Kidney Dis.

[15] Zamora E, Lupón J, de Antonio M (2014). Long-term prognostic value for patients with chronic heart failure of estimated glomerular filtration rate calculated with the new CKD-EPI equations containing cystatin C. Clin Chem.

[16] Shlipak MG, Matsushita K, Ärnlöv J (2013). Cystatin C versus creatinine in determining risk based on kidney function. N Engl J Med.

[17] Hemmelgarn BR, Zhang J, Manns BJ (2006). Progression of kidney dysfunction in the community-dwelling elderly. Kidney Int.

[18] Quiñones J, Hammad Z (2020). Social determinants of health and chronic kidney disease. Cureus.

